# Cutaneous metastasis of transitional cell carcinoma of the urinary bladder eight years after the primary: a case report

**DOI:** 10.1186/s13256-015-0585-9

**Published:** 2015-05-06

**Authors:** Andrea Nicole Lees

**Affiliations:** Royal Hobart Hospital, 48 Liverpool Street, Hobart, Tasmania 7000 Australia

**Keywords:** Cutaneous, Metastases, Transitional cell, Bladder, Urothelial, Cancer, Carcinoma

## Abstract

**Introduction:**

Cutaneous metastasis of bladder carcinoma is extremely rare with a limited number of published cases. An awareness of this rare clinical entity and high index of suspicion is needed for diagnosis, as it can occur months or rarely as in this case, even years, after the primary cancer.

**Case presentation:**

An 81-year-old Caucasian man presented with a one-year history of increasing left leg swelling and a two-month history of a macular-nodular rash on the anterior thigh, on a background of a high-grade (WHO Grade 2 of 3) papillary and invasive transitional cell carcinoma of the bladder in 2006. Following investigations, he was diagnosed as having probable locoregional recurrence of previously resected urothelial cancer of the bladder with extensive retrograde lymphatic permeation into the left thigh with cutaneous eruptions of malignancy. He completed a planned course of palliative radiation therapy to the left thigh lesions (30Gy divided over 10 fractions) as well as the left pelvic node (a total dose of 18Gy divided over six fractions). The disease ran an aggressive course and our patient died six months after the diagnosis of cutaneous metastases.

**Conclusions:**

Metastatic disease should always be considered in the differential diagnosis in patients with a previous history of bladder cancer who present with cutaneous nodules, even many years after the initial diagnosis at the primary site.

## Introduction

In 2009, there were 2,316 new cases of bladder cancer in Australia, accounting for 2% of all new cancers [1]. Bladder cancer is four times more common in men than women [1]. However, in 2010, relative survival rates for bladder cancer were higher for men than for women in Australia [2]: the five-year relative survival for bladder cancer for the period 2006 to 2010 was 60.0% for men and 49.6% for women [2]. Approximately 50% of the patients will develop local recurrence and/or metastatic disease after radical cystectomy [3]. Cutaneous metastasis of transitional cell carcinoma (TCC) is rare. The incidence is reported to be less than 1%, and ranges from 0.18% to 2% for TCC of the urinary bladder [4]. Of the cutaneous metastasis from genitourinary organs, TCC of the bladder accounts for 17% of cases [5]. It may occur at the time of diagnosis, or appear months or even years after the primary lesions are found [6]. Rarely, it may present as the first symptom of the disease [7]. We present the case of a patient with a rare cutaneous metastasis of TCC in the absence of generalized recurrent disease.

## Case presentation

An 81-year-old Caucasian man presented with a one-year history of increasing left leg swelling, initially in the calf and subsequently extending to the thigh. The general practitioner requested a venous Doppler ultrasound of the left leg and a computed tomography (CT) scan of the abdomen and pelvis, which failed to discover any underlying cause. He had developed multiple lesions on the left anterior thigh, which increased significantly in number and size over two months.

Eight years before this presentation, in 2006, he underwent a cystectomy and ileostomy for high-grade TCC of the bladder. The cystoprostatectomy specimen showed invasive TCC extending into the inner half of his muscularis propria (Stage pT2a) but with a single focus of advential intralymphatic tumor, arising in a background of high-grade transitional cell carcinoma *in situ* mainly involving the left bladder wall. Ureteric and urethral margins were clear and there was no significant prostatic pathology. Histopathology of the resected trigone and lateral bladder wall was graded 2 of 3 in the WHO classification. An abdominal and pelvic CT scan demonstrated no para-aortic or pelvic lymphadenopathy, and no distant metastasis. It was hoped that the cystoprostatectomy was curative and, therefore, he did not require adjuvant treatments.

**Figure 1:**
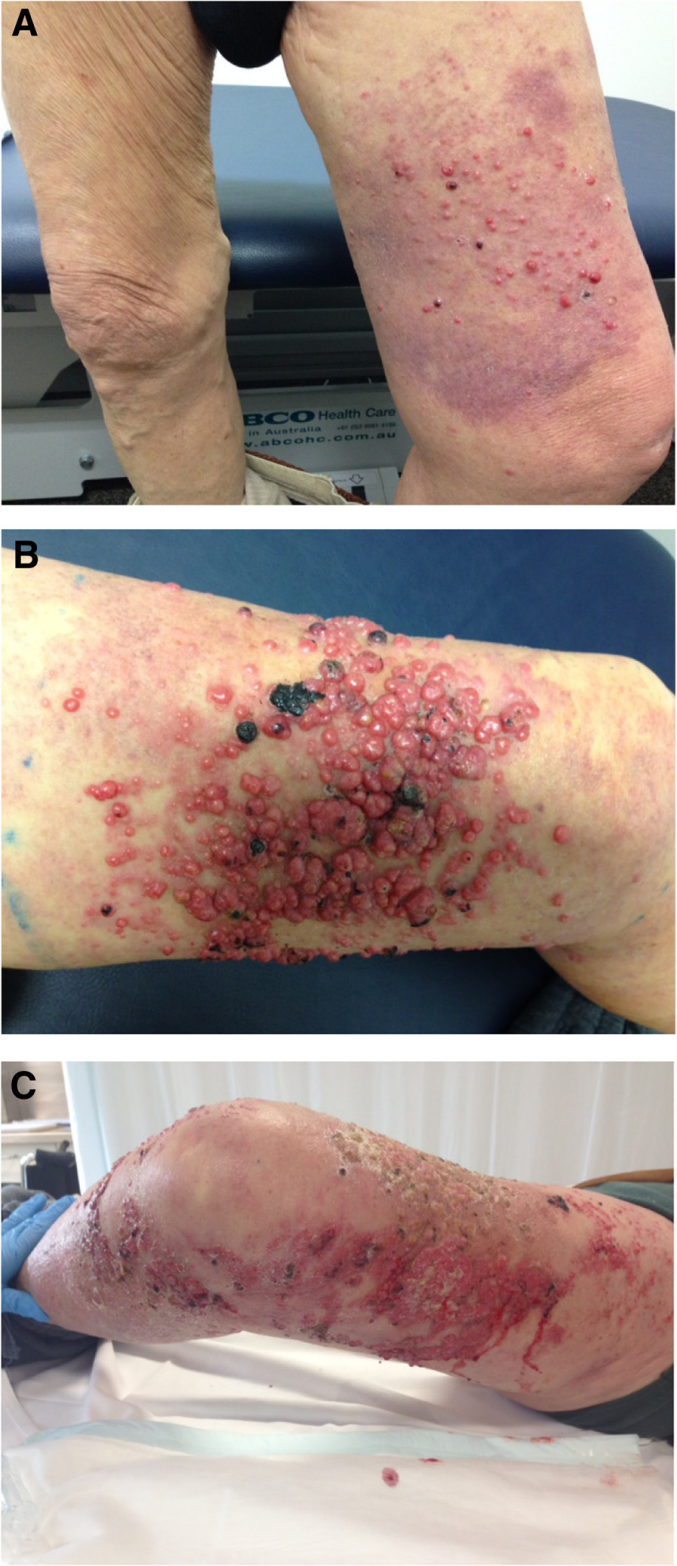
Photographs of left thigh cutaneous metastases. **(A)** At presentation; **(B)** one month later; **(C)** three months later, post radiotherapy to left leg.

On physical examination in 2014, there was left lower limb edema extending to his thigh and a well-demarcated macular-nodular rash on his anterior thigh (Figure 1A). There were no palpable masses or nodes in his groin or popliteal fossa bilaterally. His abdomen was soft and non-tender.

**Figure 2:**
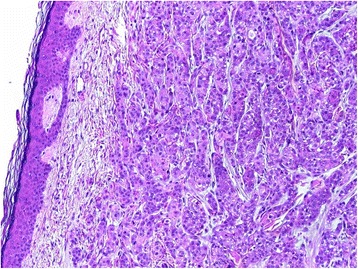
Histology showing extensive infiltration from a high-grade urothelial carcinoma (hematoxylin and eosin stain, ×100).

**Figure 3:**
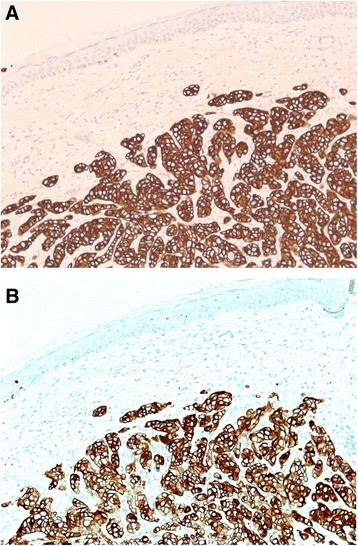
Photomicrograph of a skin punch biopsy. **(A)** Cytokeratin 7 and **(B)** cytokeratin 20.

Laboratory parameters demonstrated a normocytic anemia with a hemoglobin level of 103g/L (reference range, 130 to 175), stable since at least 2011; and normal platelet and white cell counts. Iron studies were consistent with anemia of chronic disease, with a normal ferritin level of 78ug/L (reference range, 30 to 310), reduced iron level of 3umol/L (reference range, 11 to 28) and saturation of 6% (reference range 15 to 50), and transferrin level at the lower limit of normal at 2.1g/L (reference range, 2.0 to 3.6). His B12 and folate levels were normal and thyroid-stimulating hormone level was reduced at 0.19mU/L (reference range, 0.5 to 5.5) (there were no T3 or T4 levels on record). His coagulation profile was normal. His electrolytes were normal, however, his creatinine level was elevated at 117umol/L, and his estimated glomerular filtration rate was reduced but stable (since at least 2012) at 50mL/min/1.73m2. Liver function and adjusted calcium tests were normal. There was no C-reactive protein, erythrocyte sedimentation rate, prostate-specific antigen (PSA) or other tumor markers on record. Cytology was normal on urinalysis. A skin punch biopsy demonstrated features favoring metastatic, poorly differentiated non-small cell carcinoma (Figure 2). Immunohistochemical staining (Figure 3) showed strong diffuse positive staining for cytokeratin (CK) 7 and cytokeratin 20 and focal positive staining for cytokeratin CK5/6 and P63. The tumor cells were negative for PSA and thyroid transcription factor-1 (TTF-1). The above cytokeratin immunoprofile is consistent with metastatic TCC showing focal squamoid differentiation. Left lower limb lymphoscintigraphy confirmed severe lymphedema in his left leg. A contrast-enhanced CT scan of his chest, abdomen, pelvis and thighs did not demonstrate any mass or adenopathy in the pelvis or left groin to explain our patient’s left leg lymphedema. There was no other evidence of metastatic disease. A magnetic resonance imaging (MRI) scan of the spine demonstrated no evidence of metastatic disease to his spine, however, a whole body bone scan found multiple osteoblastic metastases throughout the bony pelvis. Positron emission tomography (PET) demonstrated diffuse left leg swelling, likely related to increased tracer activity in a 17mm pelvic lymph node in the left pelvic wall. There were multiple small foci of nodular uptake seen on the anteromedial aspect of the left distal thigh consistent with the known cutaneous metastases. Sclerotic bone lesions with increased uptake on the bone scan were not highly fluorodeoxyglucose (FDG)-avid in comparison to the avid pelvic node and thought to be possibly related to an alternative pathology such as prostate cancer.

He completed a planned course of palliative radiation therapy to the left thigh lesions (30Gy divided over 10 fractions) as well as the left pelvic node (a total dose 18Gy divided over six fractions). Methotrexate was excluded as a treatment option in our patient in light of the potential for accumulation in the ‘third space’ comprising the lymphedema in his left leg. Our patient became too unwell to undergo a subsequent planned course of chemotherapy (carboplatin and gemcitabine). The disease ran an aggressive course and our patient died six months after the diagnosis of cutaneous metastases.

## Discussion

Since the first recorded case of cutaneous metastases of TCC of the urinary bladder in 1909 [8], infrequent reports have reached the world literature. Skin metastases may occur at any time after the initial diagnosis at the primary site [9]. From the published literature, cutaneous metastases most often occur within 18 months of the primary diagnosis, and only one documented case occurring 10 years after the primary diagnosis [10]. The incidence of metastatic TCC of the bladder is directly related to depth of penetration of the bladder wall, tumor grade and tumor size, with depth of tumor penetration being the single most important factor in predicting prognosis in TCC [11,12]. However, metastases may be associated with superficially invasive primary disease [13,14]. Urologic malignancies most commonly metastasize to regional lymph nodes, liver, lung and bones [5,15]. Metastatic infiltration of the skin or subcutaneous tissues can occur due to direct tumor invasion, hematogenous or lymphatic spread, or as a result of iatrogenic implantation of tumor cells [5]. Gross appearance of cutaneous metastases is not distinctive and may mimic many common dermatologic disorders [16,17]. These lesions can be solitary or multiple in appearance [14,15,18]. Brownstein *et al*. described three clinical features of metastatic cutaneous lesions, including a nodular type, inflammatory type, and sclerodermoid type [19]. In addition, a rarer zosteriform lesion has also been documented [20-22]. Metastatic skin lesions from genitourinary TCC are reported to be always located on the head, face, neck, trunk, abdomen, suprapubic region or extremities, as well as occasionally on scrotal skin and the ocular region [9].

Diagnosis requires clinical suspicion of metastases as well as histological evaluation. Diagnosis is usually established by microscopic examination of excisional biopsy specimens [9]. Among reported cases, cutaneous metastases of TCC almost exclusively show high-grade differentiation of histological grading at the primary genitourinary sites [9]. Wang *et al*. discovered that coordinated expression of cytokeratins 7 and 20 are positive in 89% of transitional cell bladder cancer [23].

The prognosis for patients with cutaneous TCC is typically poor, with a median survival of fewer than 12 months [17]. However, very rare cases of extended survival (up to 23 years) have been reported [24]. The treatment of choice for metastatic bladder cancer is either chemotherapy, with the combination of gemcitabine and cisplatin or the methotrexate, vinblastine, doxorubicin, and cisplatin (MVAC) scheme; or palliative care [25]. Chemotherapy has reported tumor remission rates up to 70% [25], but survival does not exceed 14 months [3]. Symptomatic patients may benefit from surgical resection of metastases in terms of tumor-related symptoms and performance status [26]. Local radiation therapy has also been reported to resolve cutaneous lesions, which did not respond to chemotherapy [17].

## Conclusions

This is a rare case of late presentation cutaneous metastasis of transitional cell carcinoma. Metastatic disease should always be considered in the differential diagnosis in patients with a previous history of bladder cancer who present with cutaneous nodules. This disease has a broad clinical impact across medicine, in particular for urologists, dermatologists, medical and radiation oncologists, general physicians, geriatricians and palliative care specialists. In many cases, as in our patient, treatment is mainly supportive and prognosis is poor. Due to its rarity and poor survival rate, management strategies have been difficult to define. Therefore, it is important for the medical community to have access to each case, through case reports, so as to advance our understanding of this particular disease.

## Consent

Written informed consent was obtained from the patient’s next of kin for publication of this case report and any accompanying images. A copy of the written consent is available for review by the Editor-in-Chief of this journal.
